# The conserved Pss exopolysaccharide is a common component of *Listeria* biofilms

**DOI:** 10.1128/jb.00153-26

**Published:** 2026-07-14

**Authors:** Aili Wang, Ahmed M. Elbakush, Oliver Trunschke, Mark Gomelsky

**Affiliations:** 1 Department of Molecular Biology, University of Wyoming 4416, Laramie, Wyoming, USA; Dartmouth College Geisel School of Medicine, Hanover, New Hampshire, USA

**Keywords:** glycoside hydrolase, detection probe, biofilm, exopolysaccharide, *Listeria*, listeriosis

## Abstract

*Listeria monocytogenes (Lm*) is a major foodborne pathogen capable of persisting on fresh produce and in food-processing environments. Although exopolysaccharides (EPSs) are recognized as central components of resilient bacterial biofilms, *Lm* biofilms have long been considered to be EPS-less or EPS-poor. We previously identified a listerial EPS, Pss, whose biosynthesis is encoded by the highly conserved *pss* operon and is stimulated by elevated c-di-GMP levels. However, the prevalence of Pss in wild-type strains remained unclear, in part, due to the lack of a specific detection probe. Here, we engineered a genetically encoded fluorescent probe, PssZ*-mS, based on a catalytically inactive variant (E72Q) of the Pss hydrolase PssZ. Structural modeling predicted that the E72Q substitution abolishes catalysis without disrupting Pss binding. PssZ*-mS bound purified Pss with high affinity (*K*_d_, 50 nM) and showed no detectable interaction with other tested EPSs, including cellulose, pectin, maltodextrin, or alginate. Using PssZ*-mS, we demonstrate that the reference wild-type strain EGDe produces substantial Pss under standard conditions in liquid minimal glucose-containing medium and on diverse abiotic surfaces relevant to food environments. A survey of *Lm* isolates revealed that Pss production is common, though not universal, among *Lm* strains and is also present in non-*monocytogenes Listeria* species. Notably, three genetically distinct strains from the infamous 2011 whole-cantaloupe listeriosis outbreak synthesize Pss. These findings challenge the prevailing view of listerial biofilms and identify Pss as a widespread component of *Lm* biofilms, underscoring the need for targeted anti-Pss strategies to enhance food safety.

**IMPORTANCE:**

*Listeria monocytogenes* persists on fresh produce and in food-processing environments despite rigorous sanitation, yet the molecular basis of this resilience remains poorly understood. We developed a specific fluorescent probe for the conserved Pss exopolysaccharide (EPS) and show that Pss is a common component of listerial biofilms. These findings challenge the long-standing view that *Listeria* forms primarily EPS-less or EPS-poor biofilms and identify Pss as an important biofilm component. By enabling direct visualization of Pss, this work provides a new tool for studying listerial biofilms and opens avenues for targeted antibiofilm strategies to improve food safety and public health.

## INTRODUCTION

*Listeria monocytogenes* (*Lm*) is a foodborne pathogen that causes listeriosis, a severe invasive disease with an exceptionally high case-fatality rate of approximately 15%–20% in the United States (1, 2). Although infections in immunocompetent individuals are often limited to self-resolving gastrointestinal symptoms, *Lm* poses a grave threat to vulnerable populations, including the elderly, immunocompromised individuals, pregnant women, and neonates (3). Beyond its human toll, listeriosis imposes annual multibillion-dollar economic costs due to food recalls, healthcare expenditures, and lost productivity (4).

*Lm* is distinguished by its remarkable ability to survive and persist in food-associated environments (5–7). This persistence was dramatically illustrated by the 2011 multistate cantaloupe outbreak that resulted in 33 deaths, one stillbirth, and more than 140 hospitalizations (8, 9). In this, one of the deadliest U.S. foodborne outbreaks of the century, *Lm* contamination, took place at a single packing facility, from which whole cantaloupes were shipped. The bacteria must have persisted on cantaloupe rinds during long-distance transport and storage for weeks or months, yet the biological mechanisms underlying this resilience remain poorly understood.

Another concern is the chronic persistence of *Lm* in food-processing facilities. Repeated listeriosis outbreaks have been traced to identical strains originating from the same sites over months or years despite rigorous sanitation protocols (10). While environmental factors and equipment or facilities design flaws contribute to this persistence, the biological traits that enable *Lm* to withstand repeated sanitation cycles remain incompletely characterized. Importantly, attempts to link strain persistence to specific genetic determinants have yielded inconsistent results (11–15), suggesting that these determinants may be encoded in the genomes of all *Lm* strains but expressed differentially. Together, these two persistence challenges—environmental survival on produce and chronic contamination of processing facilities—highlight critical gaps in our understanding of molecular mechanisms of *Lm*’s resilience.

Bacterial resilience on surfaces is generally associated with the biofilm lifestyle. Exopolysaccharides (EPSs) are common components of the extracellular biofilm matrix and are recognized for their contribution to biofilm stability and enhanced tolerance to chemical and physical stresses. By retaining water, forming diffusion barriers, binding antimicrobial compounds, stabilizing local microenvironments, and promoting physiological heterogeneity within biofilms, EPSs mitigate the effects of desiccation, disinfectants, antimicrobial agents, and other environmental stressors (11, 12, 16–18). Despite the well-established importance of EPSs in biofilm resilience, *Lm* has long been considered to lack substantial EPS production capacity. Its biofilm architecture has been attributed primarily to extracellular DNA, proteins, and cell-wall-associated lipoteichoic acids (19–21).

Our previous work questioned this view by demonstrating that *Lm* reference strain EGDe synthesizes copious amounts of EPS when intracellular levels of the second messenger cyclic dimeric GMP (c-di-GMP) are elevated (22). In that study, EPS synthesis was induced either by overexpression of a heterologous c-di-GMP synthase (diguanylate cyclase) or by deletion of three c-di-GMP hydrolases (phosphodiesterases), *ΔpdeB/C/D* (22). We deciphered the unique structure of the cell-associated *Lm* EPS, which we designated Pss, as a polymer of a repeating trisaccharide unit of (-4)-β-ManNAc-(β1-4)[Gal-(α1-6)]-ManNAc-(β1-), where ManNAc denotes N-acetylmannosamine and Gal denotes galactose (23). Notably, Pss does not promote attachment to polystyrene microtiter plates or other man-made materials typically used in biofilm assays (24), which differentiates it from EPSs made by major pathogens, such as PNAG in *Staphylococcus* and *Escherichia coli* (25, 26), cellulose in *E. coli* and *Salmonella enterica* (27), or Pel and Psl in *Pseudomonas aeruginosa* (28). This property may explain, in part, why Pss has been overlooked in the biofilms formed on the manmade materials despite the incredibly high conservation of the *pssA–E* operon (*lmo0527-0531*) encoding Pss biosynthesis across hundreds of sequenced clinical and environmental *Lm* isolates (22). Furthermore, the vast majority of non-*monocytogenes* species of *Listeria* also contain the *pssA–E* operon. Such conservation suggests that Pss plays an important role in listerial biology.

In the *Lm* EGDe strain with elevated c-di-GMP levels that overproduces Pss, *ΔpdeB/C/D* (hereafter strain 3Δ), Pss markedly enhances *Lm* tolerance to environmental challenges, compared to the strain impaired in Pss synthesis, *ΔpdeB/C/D ΔpssC* (hereafter strain 4Δ), conferring ~10-fold greater tolerance to desiccation, ~10¹- to 10²-fold higher tolerance to acid stress, ~10²-fold increased survival under oxidative stress, and ~10²- to 10⁶-fold higher tolerance to disinfectants compared to nonproducing strains (22, 24). These findings implicate Pss as a potentially overlooked contributor to *Lm* survival in food-associated environments. While Pss contributes significantly to environmental stress tolerance in strains with high c-di-GMP levels, it has remained uncertain whether Pss is synthesized by wild-type strains without artificially increased c-di-GMP. A major obstacle to defining the role of Pss has been the lack of a specific probe for its detection.

In the present study, we developed and characterized a fluorescent probe highly specific to Pss based on an enzymatically inactive variant of the Pss EPS hydrolase, PssZ, that we characterized previously (23). Using this probe, we examined Pss production in the reference strain, EGDe, under standard laboratory conditions and assessed the prevalence of Pss across multiple *Listeria* strains, including outbreak *Lm* isolates and selected non-*monocytogenes* strains. Our findings call for a reassessment of the role of Pss EPS in *Lm* biofilms and its implications for food safety and public health.

## RESULTS

### Design of the *Lm* Pss-specific probe

We previously described PssZ (Lmo1913), a membrane-bound, extracellular-facing glycoside hydrolase involved in Pss hydrolysis. We also engineered and characterized the PssZ E72Q variant, which is impaired in hydrolytic activity due to a mutation in the catalytically critical E72 residue (23). We hypothesized that the E72Q substitution would not impair the Pss-binding capacity of PssZ and, therefore, sought to use this mutant as a scaffold for engineering a Pss-specific probe.

**Fig 1:**
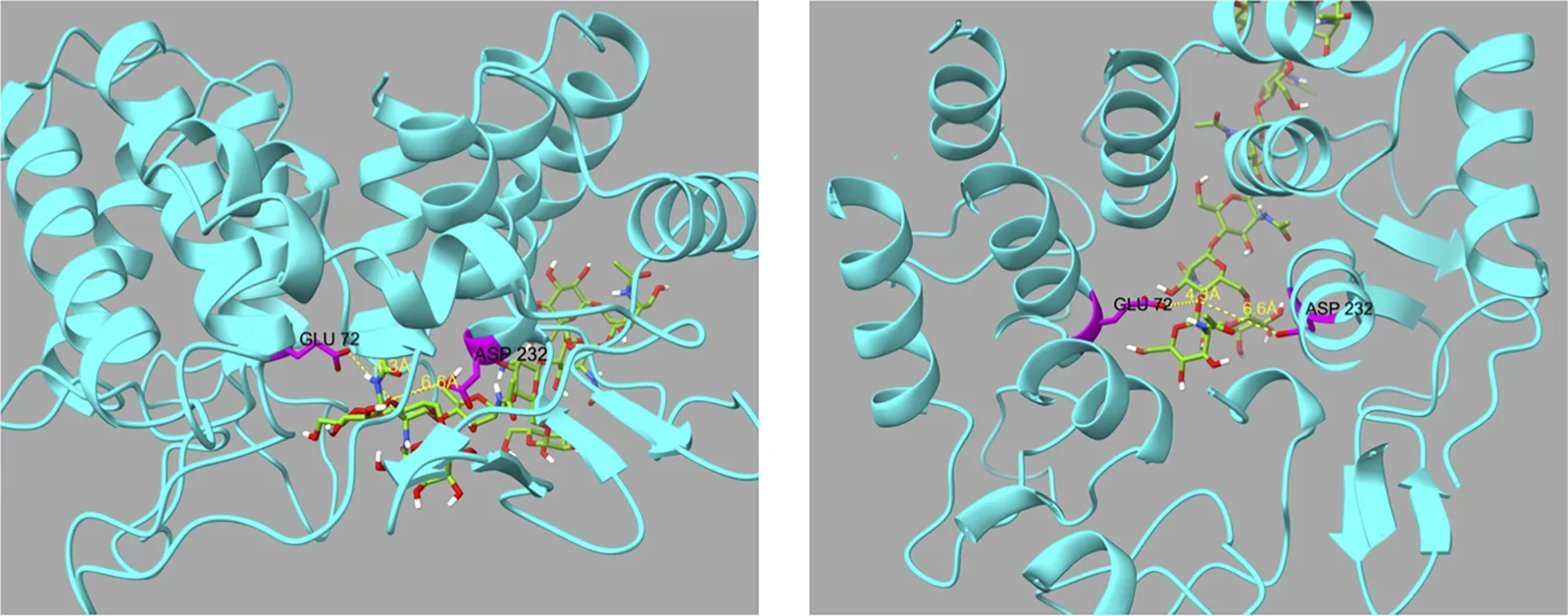
A model of the PssZ binding to the Pss polymer. The model was generated by docking a trimer of the Pss repeating unit, (-4)-β-ManNAc-(β1-4)[Gal-(α1-6)]-ManNAc-(β1-), modeled using GLYCAM-Web (31), onto PssZ structure predicted by AlphaFold 3. Docking was performed using Vina-Carb software (32). Shown are two views of one of several Pss conformers predicted to bind PssZ with the most favorable Δ*G* (–5.9 kcal/mol). Residues critical for catalysis, Glu72 and Asp232, are shown in magenta. Distances from these residues to the nearest ManNAc-(β1-4)-ManNAc glycoside bond are shown in yellow.

To assess how E72Q might affect Pss binding, we modeled PssZ–Pss interactions using an AlphaFold3-generated 3D model of the PssZ protein (29). This model is likely highly accurate, given that the X-ray structure of PssZ was previously determined (30). For Pss, we generated conformational models of the monomeric, dimeric, and trimeric repeating units of the Pss tri-saccharide using GLYCAM-Web (31) and analyzed their interactions with PssZ using AutoDock Vina-Carb (32). Models based on tri-saccharide monomers and dimers identified several potential binding sites within PssZ but did not provide a clear picture of how PssZ might interact with the Pss chain. In contrast, modeling with a trimer of the repeating unit revealed a structure in which the Pss chain is positioned within the catalytic pocket of PssZ. This model predicts that PssZ binds a trimeric unit of the Pss monomer with a favorable free energy change (Δ*G*, –5.9 kcal/mol) (Fig. 1).

The E72 residue is predicted to coordinate a water molecule critical for hydrolysis of the glycosidic bond between β-ManNAc residues. It is positioned approximately 4.3 Å from the target bond, within the typical range (~3.5–5.5 Å) observed for GH126 glycoside hydrolases to which PssZ belongs (33, 34). Similarly, the second predicted catalytic residue, D232, located 6.6 Å from the target bond, is positioned consistently with the active-site geometry of GH126 hydrolases, which we interpret as validation of the structural model. Importantly, the E72Q substitution is predicted not to alter the binding free energy of Pss (Fig. 1), supporting our strategy of using PssZ E72Q as a scaffold for engineering a Pss-specific probe.

### Testing of the Pss-specific probe in Pss detection

**Fig 2:**
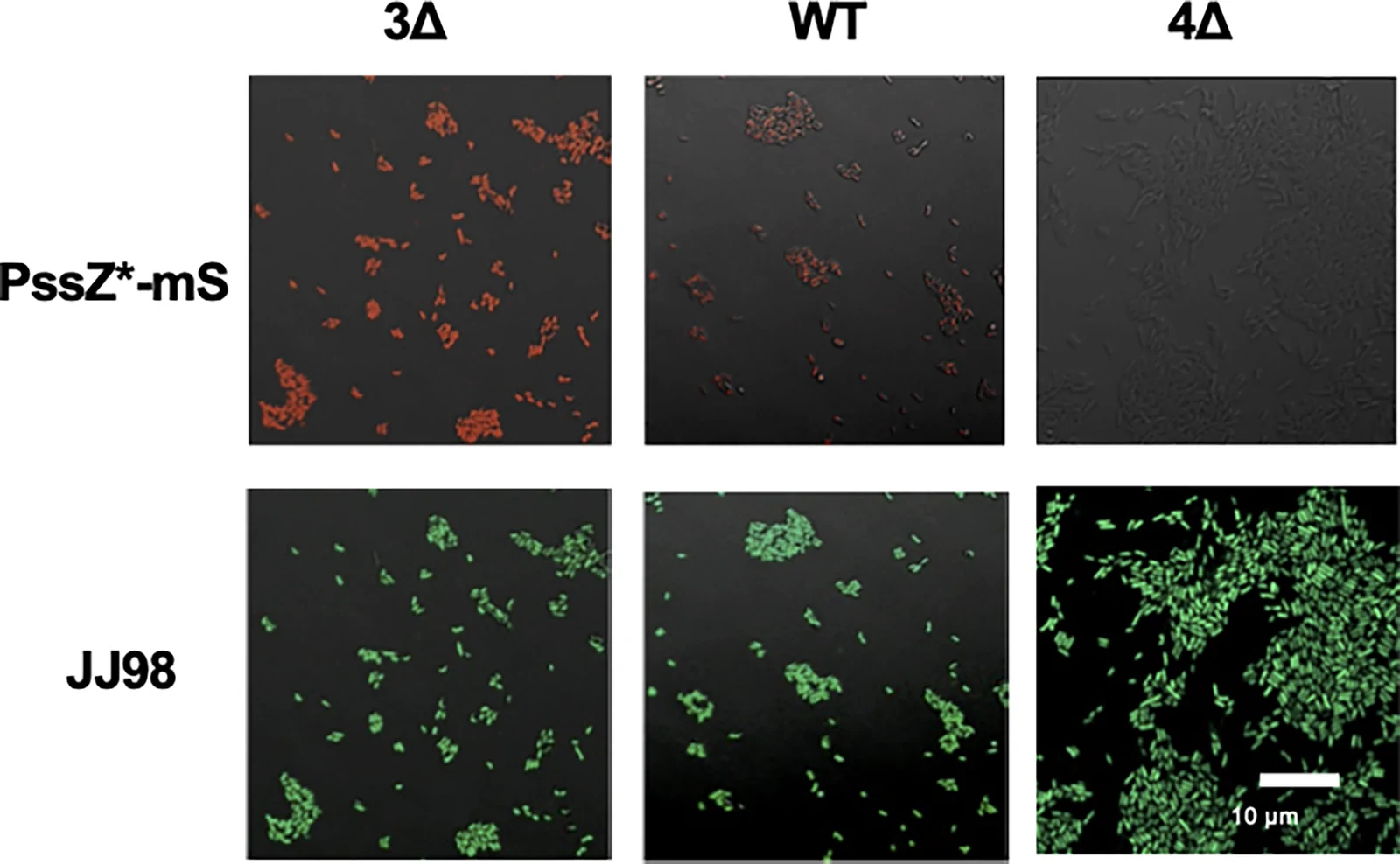
Detection of the *Lm* cell-associated Pss. Confocal fluorescence microscopy of *Lm* cells grown in liquid minimal medium. Pss (red; PssZ*-mS) and DNA (green; MycoLight Green JJ98) were visualized in the red and green fluorescence channels, respectively. Red fluorescence signal is detectable in 3Δ and the wild-type EGDe (WT) but not in the Pss-deficient strain 4Δ.

To test whether the PssZ E72Q variant could serve as a Pss-specific probe, we fused the gene encoding the soluble fragment lacking the N-terminal transmembrane domain (hereafter PssZ*) to the gene encoding the red fluorescent protein mScarlet and a His₆ tag (hereafter PssZ*-mS). We overexpressed the gene in *E. coli* BL21[DE3] and purified the fusion protein as previously described (23). PssZ*-mS binding was first assessed using the Pss-overproducing strain 3Δ. As a negative control, we used the strain 4Δ that also has high c-di-GMP levels but is impaired in Pss synthesis due to the lack of the PssC glycosyltransferase (23). MycoLight Green JJ98 was used to visualize bacterial DNA. As expected, PssZ*-mS bound to cells of the 3Δ strain but not to the Pss-deficient 4Δ strain (Fig. 2). Unexpectedly, a fraction of the wild-type EGDe cells also exhibited red fluorescence signal (Fig. 2). These results indicate that PssZ*-mS specifically detects cell-associated Pss and further suggest that EGDe produces some Pss when grown in liquid minimal medium with glucose as the carbon source.

**Fig 3:**
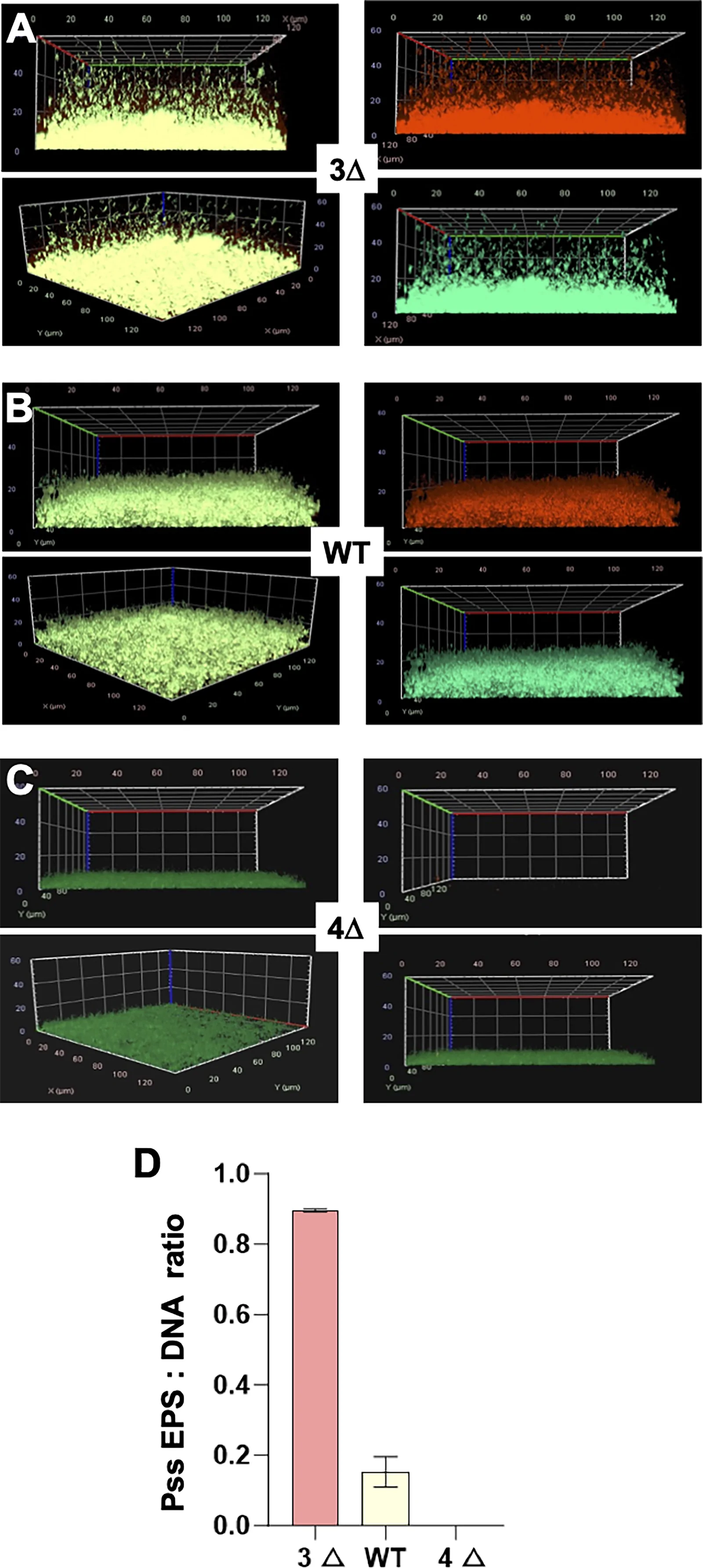
Three-dimensional architecture of *Lm* biofilms. Confocal laser scanning microscopy and 3D reconstruction of biofilms formed on glass-bottom microtiter plates coated with poly-L-lysine. (**A**) The Pss-overproducing strain 3Δ; (**B**) WT (EGDe); (**C**) the Pss-deficient strain 4Δ. Bacterial cells were stained with PssZ*-mS (red) and the DNA-staining dye MycoLight Green JJ98 (green). For each strain, four representative views are shown: upper left, dual-channel side view; lower left, dual-channel 45° tilted view; upper right, PssZ*-mS channel (Pss EPS); lower right, JJ98 channel (cells). (**D**) Pss quantification in biofilms. Three-dimensional image stacks were analyzed using Fiji with the 3D Object Counter plugin to measure total fluorescence intensity in the PssZ*-mS (EPS) and JJ98 (DNA) channels. The Pss EPS/DNA red/green fluorescence ratio was calculated to estimate relative Pss abundance normalized to bacterial biomass. The Pss EPS/DNA ratio in 3Δ, 0.92 ± 0.21, was approximately sixfold higher than the ratio in WT, 0.16 ± 0.03. Data represent mean ± SD from three independent biofilm images.

Next, we examined the presence of Pss within *Lm* biofilms by confocal laser scanning microscopy followed by three-dimensional reconstruction of biofilms. The Pss-overproducing strain 3Δ, EGDe, and the Pss-deficient strain 4Δ were grown for 2 days in liquid minimal medium supplemented with glucose in glass-bottom microtiter plates coated with poly-L-lysine. We observed robust biofilms in both 3Δ and EGDe (Fig. 3A and B). In 3Δ, in many images, Pss was seen extending well above the dense cellular layer. In contrast, EGDe biofilms exhibited a more homogeneous distribution of cells within the Pss-rich extracellular matrix. PssZ*-mS signal was not detected in strain 4Δ (Fig. 3C). Quantitative analysis of fluorescence signals showed that the ratio of Pss to DNA fluorescence in 3Δ was approximately sixfold higher than in the wild type (Fig. 3D), indicating that under static conditions, the 3Δ strain produces about six times more Pss EPS per cell than the wild type. Together, these results demonstrate that PssZ*-mS enables quantitative fluorescence analysis of Pss abundance within *Lm* biofilms.

### Ubiquity of Pss in biofilms formed by strain EGDe on diverse abiotic surfaces

**Fig 4:**
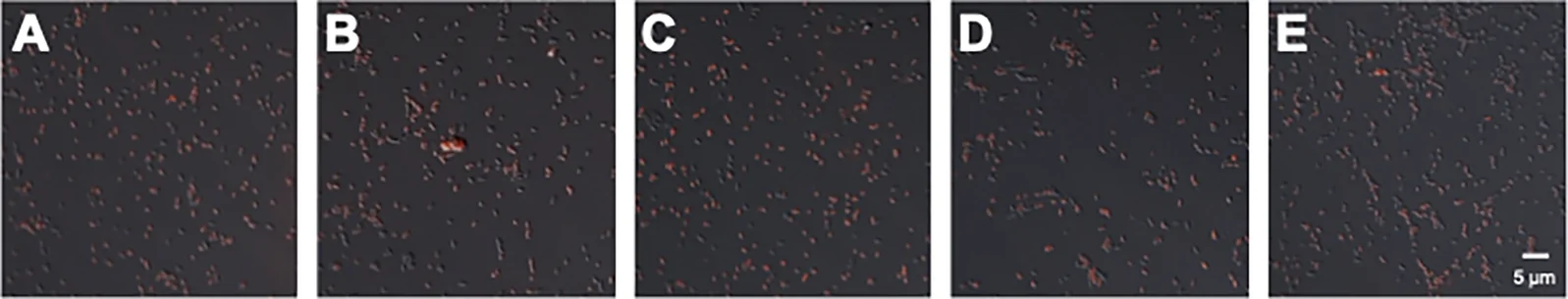
Pss production by *Lm* during growth on diverse surfaces. Single-cell confocal fluorescence microscopy of wild-type *Lm* cells recovered from biofilms formed on different abiotic materials: (**A**) wood, (**B**) polystyrene, (**C**) aluminum, (**D**) steel, and (**E**) glass. Cells were stained with PssZ*-mS (red). Pss-associated fluorescence was detected in cells from biofilms formed on all tested surfaces, indicating that wild-type *Lm* synthesizes Pss across a broad range of materials. Scale bar, 5 µm.

Given the prevailing view that *Lm* biofilms lack EPS or are EPS-poor, we asked whether Pss production by EGDe is restricted to biofilms formed on specific surface types. To address this question, we examined biofilms formed by EGDe on a range of abiotic materials relevant to food-associated environments, including stainless steel, aluminum, glass, and plastic. We also included wood, which we previously identified as material that attracts Pss-expressing cells (24). *Lm* was grown in minimal liquid medium under static conditions for 4 days in the presence of these substrates, and then biofilms were scraped, harvested, resuspended, and analyzed by confocal fluorescence microscopy using the PssZ*-mS probe. Single-cell imaging revealed Pss-associated fluorescence associated with cells from biofilms formed on all tested materials (Fig. 4). These findings demonstrate that EGDe synthesizes Pss across a broad spectrum of surfaces commonly encountered in food storage and processing environments. Thus, Pss production is not restricted to a particular substrate but appears to be a general feature of EGDe biofilms.

### Pss is a common but not ubiquitous component of biofilms across *Listeria* strains

We next assessed the prevalence of Pss production across a panel of *Listeria* strains grown in liquid glucose-containing minimal medium. The panel included genetically distinct *Lm* isolates from the infamous 2011 cantaloupe-associated listeriosis outbreak (8), *Lm* isolates from animal and human tissues, and selected nonpathogenic environmental *Listeria* species (Table 1).

**TABLE 1:** List of bacterial strains and plasmids used in this study

Strains and plasmids	Serovar and/or description	Reference or source
*L. monocytogenes* strains		
EGDe 3Δ 4Δ EGD Vitek 12,916 Li 23 Li 2 Li 2109	1/2a or 1/2c (reference strain) EGDe *ΔpdeB/pdeC/pdeD* EGDe *ΔpdeB/pdeC/pdeD ΔpssC* 1/2a (reference strain) 1/2b (quality control strain) 4a 4b 4e	ATCC BAA-679 (22) (23) ATCC 19111 ATCC BAA-751 ATCC 19114 ATCC 19115 ATCC 19118
L2625 L2626 L2676	1/2a (2011 cantaloupe outbreak) 1/2a (2011 cantaloupe outbreak) 1/2a (2011 cantaloupe outbreak)	Knabel, (35) Knabel, (35) Knabel, (35)
Nonpathogenic *Listeria* strains		
*Listeria fleischmannii* subsp. *coloradensis*		ATCC BAA-2414
*Listeria grayi*		ATCC 19120
*Listeria innocua*		ATCC 33090
*E. coli* strains		
DH10β	Strain for cloning	New England Biolabs
BL21[DE3]	Strain for protein overexpression	New England Biolabs
Plasmids		
pET23a	Vector for T7-inducible His6-fusion protein overexpression	Novagen
pET23a::PssZ*-mS	*pssZ(E72Q)::mScarlet::His_6_*	This study

**Fig 5:**
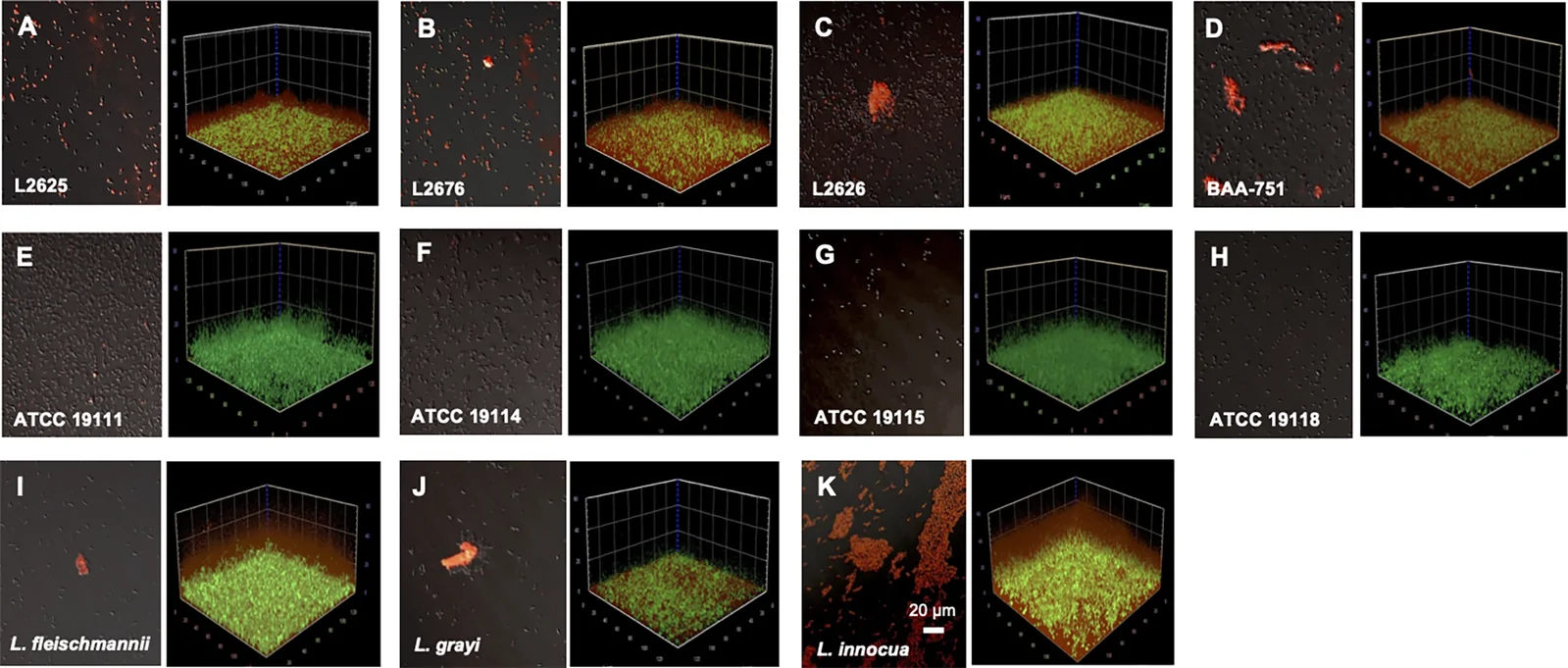
Heterogeneous Pss synthesis among c and nonpathogenic *Listeria* strains. Single-cell confocal fluorescence microscopy of *Listeria* cells grown in liquid culture and representative z-stack 3D confocal images of static biofilms formed on poly-L-lysine-coated glass slides. Pss (red; PssZ*-mS) and DNA (green; MycoLight Green JJ98) were visualized in the red and green fluorescence channels, respectively. Total red and green fluorescence signals in each channel were quantified as integrated intensities within the same segmented biofilm regions. The Pss EPS:DNA fluorescence ratio (red:green) for the imaged region is indicated in parentheses. (**A**) *Lm* L2625 (0.19), (**B**) *Lm* L2676 (0.08), (**C**) *Lm* L2626 (0.10), (**D**) *Lm* ATCC BAA751 (0.11), (**E**) *Lm* ATCC 19111 (EGD; 0), (**F**) *Lm* ATCC 19114 (0), (**G**) *Lm* ATCC 19115 (0), (**H**) *Lm* ATCC 19118 (0), and nonpathogenic strains: (**I**) *L. fleischmannii* subsp. *coloradensis* (0.42), (**J**) *L. grayi* (0.03), (**K**) *L. innocua* (1.15).

Among the cantaloupe-associated outbreak *Lm* isolates, Pss-associated fluorescence was detected on the surface of individual cells in two strains (L2625 and L2676) (Fig. 5A and B) and within spontaneously formed cell aggregates in strain L2626 (Fig. 5C). Strain BAA-751, unrelated to the 2011 outbreak, also showed Pss-fluorescence in aggregates (Fig. 5D). Several additional *Lm* strains (ATCC19111, 19114, 19115, 19118) showed no PssZ-mS* fluorescence in either aggregates or individual cells (Fig. 5E through H). In three selected nonpathogenic *Listeria* species, *Listeria fleischmannii* subsp. *coloradonensis*, *Listeria grayi,* and *Listeria innocua*, PssZ*-associated fluorescence was associated with either cell aggregates or individual cells (Fig. 5I, J and K). Importantly, all strains whose cells exhibited detectable PssZ*-mS fluorescence in liquid cultures also formed Pss-containing biofilms on poly-L-lysine-coated glass under static conditions. Among the Pss-producing strains, the relative abundance of Pss normalized to cellular DNA varied widely (Fig. 5).

Together, these results indicate that Pss synthesis is common, though not universal, among *Lm* strains, which challenges the notion that *Lm* forms primarily EPS-less or EPS-poor biofilms. Notably, the three tested 2011 outbreak strains synthesize Pss, which may have contributed to their persistence on cantaloupe rinds during lengthy fruit transportation and storage. Furthermore, the capacity for Pss synthesis is not restricted to *Lm* but extends to other members of the *Listeria* genus.

### Characterization of PssZ*-mS specificity

**Fig 6:**
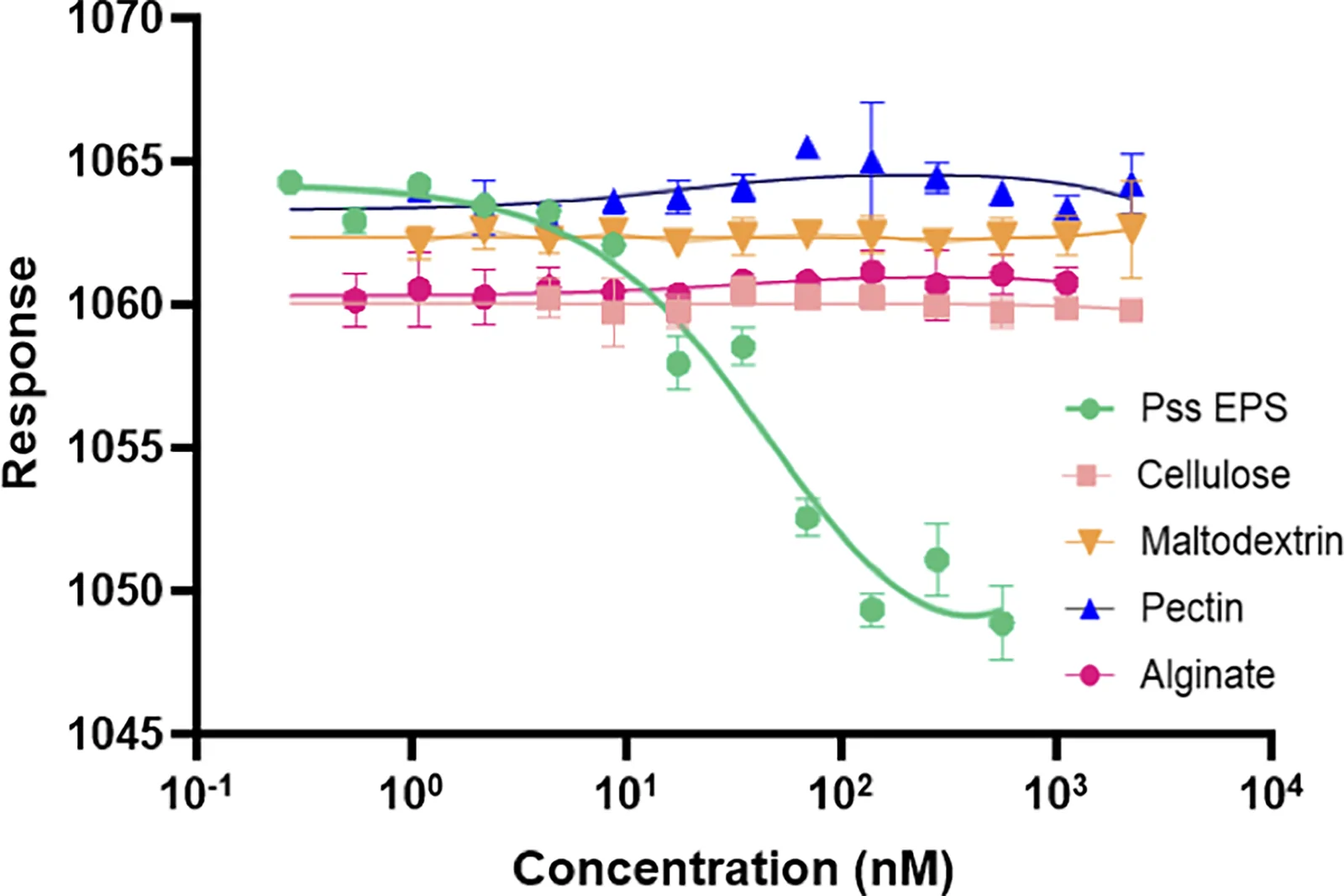
PssZ*-mS interactions with Pss. MST was used to quantify interactions between the PssZ*-mS probe and various EPSs. PssZ*-mS was titrated with increasing concentrations of listerial Pss EPS (green), cellulose (orange), maltodextrin (pink), pectin (blue), or alginate (red). A clear concentration-dependent binding response was observed only for Pss EPS, with an apparent dissociation constant, *K*_d_, 50.3 nM (95% confidence interval: 27.0–94.6 nM, determined by nonlinear regression fitting to a one-site binding model). No detectable binding was observed for the other polysaccharides, indicating high specificity of PssZ*-mS for listerial Pss. Data points represent mean ± SD from three independent measurements.

To assess the specificity of PssZ*-mS for Pss relative to other EPSs, we performed microscale thermophoresis (MST) assays (36, 37). Pss EPS for these assays was purified as described previously (23). A panel of commercially available EPSs, including cellulose, pectin, maltodextrin, and alginate, was used to evaluate specificity of Pss binding. MST analysis revealed a concentration-dependent interaction between PssZ*-mS and Pss with an apparent dissociation constant, *K*_d_, 50.3 nM. In contrast, no detectable binding was observed with any of the other EPSs tested (Fig. 6). These results demonstrate that PssZ*-mS binds *Lm* Pss with high affinity and specificity, thereby supporting its use as a selective probe for Pss detection.

## DISCUSSION

This study was motivated by the desire to reconcile two seemingly contradictory observations. On the one hand, (i) the *pss* operon encoding Pss EPS biosynthesis proteins shows extraordinary conservation across listerial genomes (22), (ii) Pss markedly enhances biofilm tolerance to various stressors (22, 24), and (iii) *Lm* persistence on fresh produce and in food-processing facilities cannot be readily explained by genetic differences among strains (11–15). On the other hand, the long-standing view holds that *Lm* biofilms lack EPS or are EPS-poor (19–21). Inspired by the development of a PNAG-specific probe based on the catalytically inactive glycoside hydrolase dispersin B from *Aggregatibacter actinomycetemcomitans* (38), we engineered a fluorescent probe, PssZ*-mS, representing a fusion of the catalytically inactive Pss hydrolase and a red fluorescent protein mScarlet (Fig. 1). The PssZ*-mS probe exhibits high affinity (*K*_d_, 50 nM) and exquisite specificity for Pss (Fig. 6) enabling the single-cell resolution imaging of Pss localization (Fig. 2, 4 and 5). Given its high affinity and specificity, PssZ*-mS is expected to facilitate detection of Pss in complex environments, including multispecies biofilms in natural settings.

We found that the reference *Lm* strain, EGDe, produces Pss under standard conditions in minimal glucose-containing medium, both in liquid culture and on various materials (Fig. 2 to 4). Notably, when Pss is overproduced, its attachment to cells appears relatively loose, as evidenced by the observation that Pss extends beyond the cell layer in static, undisturbed biofilms (Fig. 3 and 5). Unlike EPSs from many pathogens, listerial Pss does not promote attachment to most abiotic surfaces, but it does enhance attachment to plant surfaces, particularly rough substrates such as cantaloupe rinds (24). Here, we further show that Pss promotes *Lm* binding to positively charged materials, such as poly-L-lysine-coated glass (Fig. 3 and 5), consistent with the presence of negatively charged acetyl groups in the N-acetyl-ManNac units of Pss (23).

Our survey of *Lm* isolates revealed that Pss production is common but not universal among strains, at least under the conditions used in our study (Fig. 5). A central question is whether the strains that did not produce Pss are capable of Pss synthesis under different conditions. Genomic sequence analysis of the *pss* operons in the nonproducing strains detected no premature stop codons or frameshift mutations and revealed that their PssA–E proteins are highly similar to the counterparts from EGDe. For example, the PssA–E proteins from strain ATCC 19111 (strain EGD), which lacks detectable Pss production, exhibit 99%–100% amino acid identity to those from strain EGDe. Furthermore, BLAST analyses show that the PssA–E proteins are highly conserved across the genomes of hundreds of sequenced *Lm* isolates.

We suggest that the variability in Pss synthesis among the *Lm* strains observed here (Fig. 5) is likely explained by one of two factors. First, regulation of Pss synthesis may genuinely differ among strains or groups of strains. For example, three out of four strains that did not produce detectable Pss (ATCC 19114, 19115, and 19118) belong to serovar 4 (Table 1). It is unclear whether this observation is biologically significant because this sample may not be representative. Furthermore, the environmental cues and regulatory factors that control Pss synthesis in *Lm* remain largely unknown. At present, the only factor known to stimulate Pss production is elevated intracellular c-di-GMP levels, as observed in other bacteria that utilize this second messenger (39, 40). Identifying these signals will be an important direction for future studies.

A second possibility is that some *Lm* isolates have lost the capacity to synthesize Pss through laboratory domestication, a well-documented phenomenon associated with prolonged strain propagation prior to deposition (41–43). Notably, all strains that exhibited Pss synthesis (BAA-751, L2625, L2626, and L2676) were deposited soon after isolation, whereas strains lacking detectable Pss (ATCC 19111, 19114, 19115, and 19118) were isolated decades before deposition in ATCC. The only possible exception is strain EGDe, which was long believed to share a common origin with ATCC 19111 (strain EGD), isolated by E.G.D. Murray in 1924 (44), but was later shown to be a separate isolate, not directly linked to EGD (45).

The observation that several *Lm* isolates produce Pss establishes it as an important, though not universal, structural component of *Lm* biofilms (Fig. 5). Notably, three strains associated with the 2011 cantaloupe outbreak produced Pss, raising the possibility that this polymer contributed to *Lm* persistence in biofilms formed on whole cantaloupes. This possibility highlights the need to examine outbreak-associated *Lm* biofilms for Pss, for example, using the PssZ*-mS probe developed here. If Pss is present—as our data suggest—it may be necessary to supplement conventional disinfection approaches with targeted anti-Pss strategies (10). At least two strategies could be employed to counteract Pss. One approach is enzymatic degradation using the PssZ hydrolase. Exogenously applied PssZ efficiently disperses *Lm* biofilms (23); however, large-scale production of this enzyme may be costly for certain applications. An alternative strategy is inhibition of *Lm* sortase A (46), an enzyme that appears to be required for anchoring Pss to the cell surface (our unpublished data). This strategy is attractive because several natural, food-grade, and inexpensive inhibitors of *Lm* sortase A, such as polyphenols derived from maple sap and green tea, have been shown to disrupt *Lm* biofilms (47, 48). These compounds could potentially be applied to food-processing equipment or fresh produce to prevent Pss-dependent *Lm* biofilms.

## MATERIALS AND METHODS

### Bacterial strains, plasmids and culture conditions

Bacterial strains and plasmids used in this study are listed in Table 1. *Listeria* strains were routinely grown in Brain Heart Infusion (BHI, Difco) medium at 37°C or in HTM minimal medium (49) containing 3% glucose at 30°C with shaking at 150 rpm. *Escherichia coli* strains were grown in LB medium at 37°C supplemented with appropriate antibiotics when required.

### Computational modeling

The PssZ protein model was generated by AlphaFold3 (29) from the PssZ sequence lacking 26 N-terminal amino acids corresponding to the transmembrane domain. The highest ranked cif file was converted to a pdb file with ChimeraX (50). Pss pdb files representing a monomer and possible rotamers of the dimers and trimers of the Pss repeating unit were generated with the online tool GLYCAM builder (31). The pdb file of PssZ and Pss units were loaded into the software AutoDockTools-1.5.7 (51) to prepare them for docking simulations and saved as pdbqt files. The grid box was established with the same software: Box coordinates: *X*: −0.449 Å, *Y*: −4.272 Å, *Z*: −0.183 Å; Box size: *X*: 126 Å, *Y*: 126 Å, *Z*: 126 Å; with default spacing 0.375, Mode number: 3, Energy range: 5, Exhaustiveness: 8. Simulations were run on Vina-Carb using the default settings (32).

### Preparation of PssZ*-mS fluorescent probe

To generate a genetically encoded fluorescent probe for Pss detection, the DNA sequence encoding the His₆-tagged red fluorescent protein mScarlet (52) was fused to the DNA sequence encoding the C terminus of PssZ(E72Q). The gene for the resulting fusion protein, designated PssZ*-mS, was expressed in *E. coli* BL21[DE3]. The PssZ*-mS protein was purified via Co^2+^ affinity chromatography as described previously for the PssZ(E72Q)-His₆ protein (23).

### Confocal microscopy

*Listeria* strains were grown overnight in BHI medium at 37°C with shaking at 150 rpm. Cells were harvested by centrifugation, resuspended in PBS, and incubated with the PssZ*-mS probe (final concentration, 10 µM) for 20 min at room temperature prior to imaging. For static biofilms, overnight cultures were diluted 1:50 into fresh HTM medium +3% glucose, and 100 µL aliquots was added to each well of an 18-well µ-Slide chamber (ibidi). Slides were incubated statically at room temperature for 2 days to allow biofilm formation. After incubation, biofilms were gently washed with PBS and incubated with the PssZ*-mS probe (final concentration, 10 µM) for 20 min at room temperature. Unbound probe was removed by washing with PBS, followed by staining with MycoLight Green JJ98 (final concentration, 2 µM) for 10 min at room temperature. Samples were washed with PBS prior to imaging. Confocal laser scanning microscopy (Zeiss LSM 980) was performed using Z-stack acquisition with a step size of 1 µm, and image processing and three-dimensional reconstruction were carried out using FIJI software (53). Fluorescence signals from the PssZ*-mS and JJ98 channels were quantified using the 3D Object Counter plugin.

Biofilms on abiotic materials were grown as described previously (24). Briefly, sterile coupons made of wood (Woodpile), stainless steel (PH Pandahall), aluminum (RPM Stamping Blanks, Rose Metal Products), acrylic (Tupalizy, PTC-Office), and glass cover slips were placed in shaking flasks containing *Lm* EGDe cultures. After 4 days, the coupons were removed, rinsed twice in sterile HTM medium to remove loosely attached cells, and the remaining attached biofilm cells were scraped into PBS. The resulting suspensions were vortexed and stained with PssZ*-mS for imaging.

### Microscale thermophoresis

Protein-EPS interactions were analyzed by microscale thermophoresis (MST) (36) using buffer containing 20 mM Tris-HCl (pH 7.5), 150 mM NaCl, 10 mM MgCl₂, and 0.05% Tween-20. PssZ*-mS (final concentration, 100 nM) was mixed with the following EPS ligands: listerial Pss (23), cellulose (Jovvily), pectin (Fit Lane Nutrition), maltodextrin (Pure Organic Ingredients), and alginate (Talsen Chemicals). Protein-ligand mixtures were incubated at room temperature for 10 min and then loaded into MST glass capillaries. Measurements were performed using a NanoTemper Monolith instrument (NanoTemper Technologies) with fluorescence detection in the red channel. Thermophoretic responses were recorded as fluorescence changes induced by a temperature gradient. Binding curves were analyzed using NanoTemper analysis software and fitted to a single-site binding model to determine apparent dissociation constants (*K*_d_) (37).

## References

[1] Freitag NE, Port GC, Miner MD. 2009. *Listeria monocytogenes* — from saprophyte to intracellular pathogen. Nat Rev Microbiol 7:623–628. doi:10.1038/nrmicro2171 19648949 PMC2813567

[2] Centers for Disease Control and Prevention (CDC). 2024. Available from: https://www.cdc.gov/listeria

[3] Koopmans MM, Brouwer MC, Vázquez-Boland JA, van de Beek D. 2023. Human listeriosis. Clin Microbiol Rev 36:e00060-19. doi:10.1128/cmr.00060-19 36475874 PMC10035648

[4] Hoffmann S, Ahn JW. 2021. Updating economic burden of foodborne diseases estimates for inflation and income growth, ERR-297. Department of Agriculture, Economic Research Service

[5] Osek J, Lachtara B, Wieczorek K. 2022. *Listeria monocytogenes* – how this pathogen survives in food-production environments? Front Microbiol 13:866462. doi:10.3389/fmicb.2022.866462 35558128 PMC9087598

[6] Marik CM, Zuchel J, Schaffner DW, Strawn LK. 2020. Growth and survival of *Listeria monocytogenes* on intact fruit and vegetable surfaces during postharvest handling: a systematic literature review. J Food Prot 83:108–128. doi:10.4315/0362-028X.JFP-19-283 31855613

[7] Yangchen J, Sarkar D, Rood L, Vaskoska R, Kocharunchitt C. 2025. *Listeria monocytogenes*: a continuous global threat in ready-to-eat (RTE) foods. Foods 14:3664. doi:10.3390/foods14213664 41227636 PMC12607602

[8] McCollum CT, Cronquist AB, Silk BJ, Jackson KA. 2013. Multistate outbreak of listeriosis associated with cantaloupe. N Engl J Med 369:944–953. doi:10.1056/NEJMoa1215837 24004121

[9] Garner D, Kathariou S. 2016. Fresh produce–associated listeriosis outbreaks, sources of concern, teachable moments, and insights. J Food Prot 79:337–344. doi:10.4315/0362-028X.JFP-15-387 26818997

[10] Ferreira V, Wiedmann M, Teixeira P, Stasiewicz MJ. 2014. *Listeria monocytogenes* persistence in food-associated environments: epidemiology, strain characteristics, and implications for public health. J Food Prot 77:150–170. doi:10.4315/0362-028X.JFP-13-150 24406014

[11] Harrand AS, Skeens J, Carroll L, Orsi R, Wiedmann M, Bolten S. 2026. *Listeria* sanitizer tolerance at use-level concentrations shows limited association with genetic loci. Appl Environ Microbiol 92:e0106025. doi:10.1128/aem.01060-25 41556918 PMC12915297

[12] Limoli DH, Jones CJ, Wozniak DJ. 2015. Bacterial extracellular polysaccharides in biofilm formation and function. Microbiol Spectr 3. doi:10.1128/microbiolspec.MB-0011-2014 PMC4657554 26185074

[13] Finn L, Onyeaka H, O’Neill S. 2023. *Listeria monocytogenes* biofilms in food-associated environments: a persistent enigma. Foods 12:3339. doi:10.3390/foods12183339 37761048 PMC10529182

[14] Maggio F, Rossi C, Chiaverini A, Ruolo A, Orsini M, Centorame P, Acciari VA, Chaves López C, Salini R, Torresi M, Serio A, Pomilio F, Paparella A. 2021. Genetic relationships and biofilm formation of *Listeria monocytogenes* isolated from the smoked salmon industry. Int J Food Microbiol 356:109353. doi:10.1016/j.ijfoodmicro.2021.109353 34411997

[15] Burdová A, Véghová A, Minarovičová J, Drahovská H, Kaclíková E. 2024. The relationship between biofilm phenotypes and biofilm-associated genes in food-related *Listeria monocytogenes* strains. Microorganisms 12:1279. doi:10.3390/microorganisms12071297 39065070 PMC11279107

[16] Flemming HC, Wingender J. 2010. The biofilm matrix. Nat Rev Microbiol 8:623–633. doi:10.1038/nrmicro2415 20676145

[17] Karygianni L, Ren Z, Koo H, Thurnheer T. 2020. Biofilm matrixome: extracellular components in structured microbial communities. Trends Microbiol 28:668–681. doi:10.1016/j.tim.2020.03.016 32663461

[18] Sauer K, Stoodley P, Goeres DM, Hall-Stoodley L, Burmølle M, Stewart PS, Bjarnsholt T. 2022. The biofilm life cycle: expanding the conceptual model of biofilm formation. Nat Rev Microbiol 20:608–620. doi:10.1038/s41579-022-00767-0 35922483 PMC9841534

[19] Borucki MK, Peppin JD, White D, Loge F, Call DR. 2003. Variation in biofilm formation among strains of *Listeria monocytogenes*. Appl Environ Microbiol 69:7336–7342. doi:10.1128/AEM.69.12.7336-7342.2003 14660383 PMC309931

[20] Renier S, Hébraud M, Desvaux M. 2011. Molecular biology of surface colonization by *Listeria monocytogenes*: an additional facet of an opportunistic Gram-positive foodborne pathogen. Environ Microbiol 13:835–850. doi:10.1111/j.1462-2920.2010.02378.x 21087384

[21] Guilbaud M, Piveteau P, Desvaux M, Brisse S, Briandet R. 2015. Exploring the diversity of *Listeria monocytogenes* biofilm architecture by high-throughput confocal laser scanning microscopy and the predominance of the honeycomb-like morphotype. Appl Environ Microbiol 81:1813–1819. doi:10.1128/AEM.03173-14 25548046 PMC4325147

[22] Chen L-H, Köseoğlu VK, Güvener ZT, Myers-Morales T, Reed JM, D’Orazio SEF, Miller KW, Gomelsky M. 2014. Cyclic di-GMP-dependent signaling pathways in the pathogenic Firmicute *Listeria monocytogenes*. PLoS Pathog 10:e1004301. doi:10.1371/journal.ppat.1004301 25101646 PMC4125290

[23] Köseoğlu VK, Heiss C, Azadi P, Topchiy E, Güvener ZT, Lehmann TE, Miller KW, Gomelsky M. 2015. *Listeria monocytogenes* exopolysaccharide: origin, structure, biosynthetic machinery and c-di-GMP-dependent regulation. Mol Microbiol 96:728–743. doi:10.1111/mmi.12966 25662512

[24] Fulano AM, Elbakush AM, Chen LH, Gomelsky M. 2023. The *Listeria monocytogenes* exopolysaccharide significantly enhances colonization and survival on fresh produce. Front Microbiol 14:1126940. doi:10.3389/fmicb.2023.1126940 37180237 PMC10172500

[25] Heilmann C, Gerke C, Perdreau-Remington F, Götz F. 1996. Characterization of Tn917 insertion mutants of *Staphylococcus epidermidis* affected in biofilm formation. Infect Immun 64:277–282. doi:10.1128/iai.64.1.277-282.1996 8557351 PMC173756

[26] Wang X, Preston JF, Romeo T. 2004. The *pgaABCD* locus of *Escherichia coli* promotes the synthesis of a polysaccharide adhesin required for biofilm formation. J Bacteriol 186:2724–2734. doi:10.1128/JB.186.9.2724-2734.2004 15090514 PMC387819

[27] Zogaj X, Nimtz M, Rohde M, Bokranz W, Römling U. 2001. The multicellular morphotypes of *Salmonella* typhimurium and *Escherichia coli* produce cellulose as the second component of the extracellular matrix. Mol Microbiol 39:1452–1463. doi:10.1046/j.1365-2958.2001.02337.x 11260463

[28] Friedman L, Kolter R. 2004. Two genetic loci produce distinct carbohydrate-rich structural components of the *Pseudomonas aeruginosa* biofilm matrix. J Bacteriol 186:4457–4465. doi:10.1128/JB.186.14.4457-4465.2004 15231777 PMC438632

[29] Abramson J, Adler J, Dunger J, Evans R, Green T, Pritzel A, Ronneberger O, Willmore L, Ballard AJ, Bambrick J, et al.. 2024. Accurate structure prediction of biomolecular interactions with AlphaFold 3. Nature 630:493–500. doi:10.1038/s41586-024-07487-w 38718835 PMC11168924

[30] Wu H, Qiao S, Li D, Guo L, Zhu M, Ma LZ. 2019. Crystal structure of the glycoside hydrolase PssZ from *Listeria monocytogenes*. Acta Crystallogr F Struct Biol Commun 75:501–506. doi:10.1107/S2053230X19008100 31282870 PMC6613446

[31] Kirschner KN, Yongye AB, Tschampel SM, González-Outeiriño J, Daniels CR, Foley BL, Woods RJ. 2008. GLYCAM06: a generalizable biomolecular force field. Carbohydrates. J Comput Chem 29:622–655. doi:10.1002/jcc.20820 17849372 PMC4423547

[32] Nivedha AK, Thieker DF, Makeneni S, Hu H, Woods RJ. 2016. Vina-Carb: improving glycosidic angles during carbohydrate docking. J Chem Theory Comput 12:892–901. doi:10.1021/acs.jctc.5b00834 26744922 PMC5140039

[33] Fujiwara T, Fujishima A, Nakamura Y, Tajima K, Yao M. 2022. Structural snapshot of a glycoside hydrolase family 8 endo-β-1,4-glucanase capturing the state after cleavage of the scissile bond. Acta Crystallogr D Struct Biol 78:228–237. doi:10.1107/S2059798321012882 35102888 PMC8805304

[34] Kerényiová L, Janeček Š. 2020. A detailed *in silico* analysis of the amylolytic family GH126 and its possible relatedness to family GH76. Carbohydr Res 494:108082. doi:10.1016/j.carres.2020.108082 32634753

[35] Lomonaco S, Verghese B, Gerner-Smidt P, Tarr C, Gladney L, Joseph L, Katz L, Turnsek M, Frace M, Chen Y, Brown E, Meinersmann R, Berrang M, Knabel S. 2013. Novel epidemic clones of *Listeria monocytogenes*, United States, 2011. Emerg Infect Dis 19:147–150. doi:10.3201/eid1901.121167 23260778 PMC3558006

[36] Wienken CJ, Baaske P, Rothbauer U, Braun D, Duhr S. 2010. Protein-binding assays in biological liquids using microscale thermophoresis. Nat Commun 1:100. doi:10.1038/ncomms1093 20981028

[37] Seidel SAI, Dijkman PM, Lea WA, van den Bogaart G, Jerabek-Willemsen M, Lazic A, Joseph JS, Srinivasan P, Baaske P, Simeonov A, Katritch I, Melo FA, Ladbury JE, Schreiber G, Watts A, Braun D, Duhr S. 2013. Microscale thermophoresis quantifies biomolecular interactions under previously challenging conditions. Methods 59:301–315. doi:10.1016/j.ymeth.2012.12.005 23270813 PMC3644557

[38] Eddenden A, Kitova EN, Klassen JS, Nitz M. 2020. An inactive Dispersin B probe for monitoring PNAG production in biofilm formation. ACS Chem Biol 15:1204–1211. doi:10.1021/acschembio.9b00907 31917539

[39] Römling U, Galperin MY, Gomelsky M. 2013. Cyclic di-GMP: the first 25 years of a universal bacterial second messenger. Microbiol Mol Biol Rev 77:1–52. doi:10.1128/MMBR.00043-12 23471616 PMC3591986

[40] Jenal U, Reinders A, Lori C. 2017. Cyclic di-GMP: second messenger extraordinaire. Nat Rev Microbiol 15:271–284. doi:10.1038/nrmicro.2016.190 28163311

[41] McLoon AL, Guttenplan SB, Kearns DB, Kolter R, Losick R. 2011. Tracing the domestication of a biofilm-forming bacterium. J Bacteriol 193:2027–2034. doi:10.1128/JB.01542-10 21278284 PMC3133032

[42] Eydallin G, Ryall B, Maharjan R, Ferenci T. 2014. The nature of laboratory domestication changes in freshly isolated *Escherichia coli* strains. Environ Microbiol 16:813–828. doi:10.1111/1462-2920.12208 23889812

[43] Steensels J, Gallone B, Voordeckers K, Verstrepen KJ. 2019. Domestication of industrial microbes. Curr Biol 29:R381–R393. doi:10.1016/j.cub.2019.04.025 31112692

[44] Murray EGD, Webb RA, Swann MBR. 1926. A disease of rabbits characterised by a large mononuclear leucocytosis, caused by a hitherto undescribed bacillus *Bacterium monocytogenes* (n.sp.). J Pathol 29:407–439. doi:10.1002/path.1700290409

[45] Bécavin C, Bouchier C, Lechat P, Archambaud C, Creno S, Gouin E, Wu Z, Kühbacher A, Brisse S, Pucciarelli MG, García-del Portillo F, Hain T, Portnoy DA, Chakraborty T, Lecuit M, Pizarro-Cerdá J, Moszer I, Bierne H, Cossart P. 2014. Comparison of widely used *Listeria monocytogenes* strains EGD, 10403S, and EGD-e highlights genomic variations underlying differences in pathogenicity. mBio 5:e00969-14. doi:10.1128/mBio.00969-14 24667708 PMC3977354

[46] Jacobitz AW, Kattke MD, Wereszczynski J, Clubb RT. 2017. Sortase transpeptidases: structural biology and catalytic mechanism. Adv Protein Chem Struct Biol 109:223–264. doi:10.1016/bs.apcsb.2017.04.008 28683919 PMC5718342

[47] Elbakush AM, Fulano AM, Gomelsky M. 2023. Lignan-containing maple products inhibit *Listeria monocytogenes* biofilms on fresh produce. Front Microbiol 14:1258394. doi:10.3389/fmicb.2023.1258394 37928682 PMC10620520

[48] Elbakush AM, Trunschke O, Shafeeq S, Römling U, Gomelsky M. 2024. Maple compounds prevent biofilm formation in *Listeria monocytogenes* via sortase inhibition. Front Microbiol 15:1436476. doi:10.3389/fmicb.2024.1436476 39351304 PMC11439720

[49] Tsai HN, Hodgson DA. 2003. Development of a synthetic minimal medium for *Listeria monocytogenes*. Appl Environ Microbiol 69:6943–6945. doi:10.1128/AEM.69.11.6943-6945.2003 14602660 PMC262277

[50] Meng EC, Goddard TD, Pettersen EF, Couch GS, Pearson ZJ, Morris JH, Ferrin TE. 2023. UCSF ChimeraX: Tools for structure building and analysis. Protein Sci 32:e4792. doi:10.1002/pro.4792 37774136 PMC10588335

[51] Morris GM, Huey R, Lindstrom W, Sanner MF, Belew RK, Goodsell DS, Olson AJ. 2009. AutoDock4 and AutoDockTools4: automated docking with selective receptor flexibility. J Comput Chem 30:2785–2791. doi:10.1002/jcc.21256 19399780 PMC2760638

[52] Bindels DS, Haarbosch L, van Weeren L, Postma M, Wiese KE, Mastop M, Aumonier S, Gotthard G, Royant A, Hink MA, Gadella TWJ Jr. 2017. mScarlet: a bright monomeric red fluorescent protein for cellular imaging. Nat Methods 14:53–56. doi:10.1038/nmeth.4074 27869816

[53] Schindelin J, Arganda-Carreras I, Frise E, Kaynig V, Longair M, Pietzsch T, Preibisch S, Rueden C, Saalfeld S, Schmid B, Tinevez J-Y, White DJ, Hartenstein V, Eliceiri K, Tomancak P, Cardona A. 2012. Fiji: an open-source platform for biological-image analysis. Nat Methods 9:676–682. doi:10.1038/nmeth.2019 22743772 PMC3855844

